# Chicago Gynæcological Society; Meeting of March 18th

**Published:** 1887-05

**Authors:** 

**Affiliations:** 2330 Indiana avenue


### TRANSACTIONS OF THE CHICAGO GYNÆCOLOGICAL SOCIETY.

*Regular Meeting, Friday, March 18th, 1887.*

*I.*—JAGGARD. *A Puerperal Uterus, Showing Endometritis Puerperalis.*

*II.*—JACKSON. *A Case of Removal of the Ovaries and Tubes for Bleeding Fibro-Myoma of the Uterus.*

*III.*—FENGER. *The Operative Treatment of Retroperitoneal Cysts in Connection with Mikulicz' Method of Drainage.*

The PRESIDENT, CHARLES WARRINGTON EARLE, M.D., in the Chair.

*I.*—DR. W. W. JAGGARD presented a

*Puerperal Uterus, showing Endometritis Puerperalis.*

The material was placed at his disposal through the courtesy of Drs. Pickard, Hibbard and Plummer, of the Cook County Hospital.

*History:* Patient 64003, Ward 14, Swede, 24 years old. I para. Labor, March 13th. Vertex presentation, R. O. P.: First stage, ten hours; second stage, three hours; third stage, ten minutes; male child; wt. 6 lbs., 13 oz.; condition good. Severe post-partum hæmorrhage, after expression of placenta; intra-uterine irrigation with hot water, vinegar, finally mercuric chloride, 1: 4000: perineum sutured, vaginal douche. Chill, three hours after delivery; temperature 101°.8 F.; pulse rapid and feeble. Patient died four days later; temperature reaching the maximum, 103° F. a few hours before death.

The autopsy disclosed puerperal ulcers in the vagina, diphtheritic endometritis, splenic tumor, ulcerative septic-endocarditis. The ovaries, tubes and peritoneum were not involved in the inflammatory process. The case apparently corresponded to the form described by Buhl in 1861 as "Puerperal pyæmia without peritonitis" (metro-phlebitis). The poison had apparently gained direct access to the veins, the endometritis being only a prominent symptom.

It must not be supposed that the mortality rate from child-bed fever in the lying-in wards at the Cook County Hospital is high. About 400 women are annually delivered in that institution, and less than one-half of one per centum perish from puerperal fever. Rigid antiseptic precautions, in conformity with the Semmelweis doctrine—every case of child-bed fever arises from the resorption of decomposing organic matter through some local lesion in the genital tract—have been instituted and carried out with commendable intelligence, skill and zeal by the internes. The only criticism that Dr. Jaggard wished to make was upon the use of mercuric chloride for the purpose of intra-uterine irrigation.

DISCUSSION.

DR. CHARLES WARRINGTON EARLE: I think there is evidence accumulating at this time which shows that we are not certain that the decidua comes away completely, and in a great number of cases there is not only a considerable amount of this left, but also pieces of membrane, etc., etc., which no one, however careful, can detect.

Whenever a high temperature takes place on the eighth or ninth or tenth day, I am in the habit of washing out the uterus, and then if the high temperature continues, to curette, and in almost every case the amount brought away astonishes everybody, particularly the attending accoucheur. Sometimes 2 or 3 drachms are brought away; the temperature becomes normal in 12 hours and a speedy convalescence takes place. I would like to ask what th eexperience and observation of the other Fellows of the Society have been in regard to the certainty which they feel that *everything* comes away.

I am getting together a series of cases in which I will endeavor to show that where there is a high temperature that comes on as late as the tenth or eleventh day, if it does not come down after a thorough intra-uterine douche with carbolized water, curetting should be resorted to, and that it will in almost every case bring away an amount of material that none of us expect. In many of these cases the temperature goes down at once.

I had a case on Hoyne street, which took place in the practice of a graduate of one of our schools,—a young man of excellent record, and one who is thoroughly in accord with us on antiseptic obstetrics. On the tenth day the temperature was 103½° F.; an intra-uterine douche did not bring it down. The next day it was 105½° F. I curetted and brought away a mass of material which surprised me as well as the practitioner. The woman made an excellent recovery without a bad symptom.

DR. A. REEVES JACKSON: It seems, to look at this specimen, that the curettement would have to be exceedingly deep; especially after the infection of the walls, you could hardly get away all the septic influence. There may have been a stage in the history of that case in which it would have been a proper treatment.

DR. CHRISTIAN FENGER: In consultation, last fall, I saw a case of abortion, not childbirth, where part of the placenta was left. The temperature had been down for over a week and the woman was apparently perfectly well. The doctor in charge decided to curette that part of the placenta which remained. The curettement was immediately followed by sepsis that terminated fatally within a short time. So it seems that under certain circumstances curettement may open up sepsis. He curetted to remove the placenta.

DR. JAGGARD said he supposed Dr. Earle referred to Carl Braun's practice of curettement. He did not care to discuss that subject at this meeting. Certainly, in the case presented curettement could have accomplished nothing. The poison—ptomaine, cadaveric poison, or whatever it was—had already entered the veins.

*II.*—DR. A. REEVES JACKSON read the following report of

*A Case of Removal of the Ovaries and Tubes for Fibro-Myoma of the Uterus.*

I present to the society a pair of ovaries and corresponding tubes, which were removed for the purpose of a possible beneficial influence on the hemorrhage caused by bleeding myoma of the uterus. The woman was single, about 40 years of age, menstruated first at the age of 13, and was always regular until three years ago, when menorrhagia occurred. At the same time, there appeared at intermenstrual periods—about half-way between—occasional discharges of a yellowish thin fluid. These discharges were preceded by pain in the left iliac region and some swelling. The menorrhagia increased, the other discharges continued about the same, and bleeding became so nearly constant that finally it was difficult to distinguish the menstrual period. About a year and a half before I saw her, there had been discovered a tumor in the abdomen. Ergot was then used systematically and properly, but it had no restraining influence on the hemorrhage and she became anemic, exhausted, and had nervous headaches, cardiac palpitation and loss of appetite. I found a uterine fibroid the size of an orange and suggested removal of the ovaries, although she came for the purpose of having the uterus removed. The operation was performed at the Presbyterian Hospital three weeks ago. The left ovary was exceedingly small with no trace of recent ovulation ; the tube was enlarged and contained a small amount of yellowish fluid. Possibly this condition of the tube, with the previous history of occasional swelling, may have indicated that the latter was from hydrosalpinx. Here are the specimens. The right ovary was enlarged and the tube normal in structure. There was a Graafian follicle which had just broken. She had menstruation two weeks before and the condition of this follicle seemed to indicate that it had no temporal relation to that menstrual period. The highest temperature was 100.1° F., and was reached on the fourth day. The woman is now entirely well, is sitting up, and will probably be able to go to her home, a hundred or two miles distant, in the coming week. Hemorrhage appeared on the third day after operation and lasted thirty-six hours. It was then checked by vaginal tampons. She has had no discharge of blood since that time. What effect the operation will ultimately have upon hemorrhage and growth of the tumor cannot be determined at this stage of the history.

*III.*—DR. CHRISTIAN FENGER made the following remarks on

*The Operative Treatment of Retroperitoneal Cysts in Connection with Mikulicz' Method of Drainage.*

It is not my intention to-night to give an exhaustive review of the entire subject of retroperitoneal or parovarian cysts, but I merely wish to call attention to the subject for discussion, giving some of my own experiences, with a view of bringing out those of other Fellows of the Society.

The subject is that of so-called parovarian cysts, or cysts of the broad ligament, or cysts with fimbriated ephithelium, and I wish to call attention to a few facts concerning them before showing specimens.

We know that these cysts are said, in a great majority of cases, to develop from the parovarium, the rudimentary sexual remnant of the Wolffian bodies; more rarely, they are said to develop from the epoöphoron; finally, it is possible that cysts of the broad ligament may originate from hæmatomas.

The canals of the parovarium, being lined with fimbriated epithelium, may account for the fact that the inside of a number of these cysts is found to be lined with this form of epithelium.

Parovarian cysts are typically mono-cysts. In this respect they differ materially from proliferating cystomas or other ovarian cysts developed in or into the broad ligament. Both classes are retroperitoneal cysts, inasmuch as they are situated behind the peritoneum of the posterior wall of the abdomen, but the cysts of ovarian origin are more likely to have only a partial retroperitoneal or intra-ligamentous development; that is, part of the tumor within, part outside of the broad ligament; whilst the parovarian cysts proper are more likely to be completely surrounded by the broad ligament. From the broad ligament, and separating its two layers, they commonly develop inward to the sides of the uterus and downward toward the bottom of the small pelvis.

They are usually thin-walled, lined with fimbriated epithelium or mixed fimbriated and common cylindrical epithelium; consequently their interior surface is smooth, and they contain a thin, colorless, clear fluid of low specific gravity, with no formed elements. Between the peritoneal covering and the cyst-wall there is usually a layer of loose connective tissue with but few vessels; which explains the facility with which these cysts may sometimes be separated from the broad ligament covering them, and enucleated without the use of cutting instruments, and with very little harm.

A typical cyst of this kind should have the Fallopian tube on its outside stretched out and flattened, because the cyst develops into the little mesentery of the tube. In the same way the ovary is found stretched out and flattened on the outside of the cyst near the tube. Exceptions to these common anatomical characters, however, are found. The cyst-wall may be thick, may become the seat of secondary growths, such as papillæ or papillomatous fimbriated tumors, which, having developed on the inside of the cyst, may perforate the cyst-wall, protrude on the outside, and take upon them a malignant or semi-malignant character, invade the general peritoneal cavity, giving rise to multiple metastatic papillomas.

In cases of this kind, the contents of the cyst is not a thin, clear, serous fluid, but resembles more or less the fluid of the ovarian cystomas, with numerous formed elements, viscid character, and hæmative or blood mixed with it.

The connective tissue layer between the cyst and the broad ligament may not be loose and deficient in vessels, but is sometimes so tense as to make separation of the cyst here almost or entirely impossible, and it may contain numerous large vessels.

As to the symptoms: The cysts usually grow slowly, and do not cause any inconvenience unless they reach a very considerable size. They are usually not very tense. The fluctuation is very distinct and superficial. When such a mono-cyst is large, the abdomen is likely to be flat when the patient is recumbent, as in ascites, and the percussion note is apt to change somewhat with the position of the patient, thereby sometimes making the differential diagnosis still more difficult.

The parovarian cysts are likely to burst spontaneously, but the contained fluid is so little irritative in character that peritonitic symptoms rarely follow, the thin, clear fluid being absorbed quickly and readily.

On this account, these are the cysts of the abdominal cavity which best permit of puncture or aspiration, as these trifling operations are not uncommonly followed by radical cures.

In this connection I will describe a case which came under my observation in 1884. A girl 18 years of age came to me from Racine, who had a cyst extending above the umbilicus, and about the size of a uterus in the seventh month of gestation. She had been accused by her relatives of being pregnant, but, knowing this was not the case, came on here.

On examination I found the uterus of normal size on one side of the cyst, and in my office, with a common hypodermic syringe, I drew off and took away for examination a perfectly clear fluid, and told the patient to come down for operation. She went home to make her arrangements, and came down a month later. The cyst had entirely disappeared, without symptoms of peritonitis.

In a case like this there may, of course, be a doubt as to the correctness of the diagnosis of a parovarian cyst; but it is reasonably certain that this was the case, as one of the characteristics of this class of cysts is that rupture into the peritoneal cavity causes no peritonitis, and the fluid is absorbed without difficulty.

The method of operating on these cysts we owe to Dr. Miner, of Buffalo, N. Y., who published in 1869 his operation by enucleation.

The surface of the tumor, or, rather, the broad ligament, when exposed after the opening of the abdominal cavity, is incised down to the wall of the cyst. In the loose connective tissue layer the broad ligament is now separated from the cyst-wall. By means of the fingers or blunt instruments this separation can be continued, without the use of any force and without appreciable hæmorrhage, until the cyst is completely enucleated, and may be lifted out of the cavity. Evacuation of the cyst fluid after partial denudation of the wall, as a matter of course, facilitates enucleation.

In some cases of parovarian cysts, the development is to such an extent peripheral in the broad ligament that the uterine half of the latter is long enough for the formation of a pedicle. In such cases the usual operation for ovarian cysts may be performed at a sacrifice of the covering broad ligament, with tube and ovary. But such a peripheral development is not the rule, and whenever the cyst is developed down upon the uterus or into Douglas' fossa, or farther away still in the retroperitoneal space, enucleation is the only method available for its complete removal.

Difficulties during the course of enucleation arise when the connective tissue is tense and rich in vessels, necessitating dissection with the knife, and numerous ligatures. Further, if a large cyst develops deep down in Douglas' fossa or even behind the rectum, or up into the mesenteries of the intestines, sigmoid flexure, or descending colon on the left side, or cæcum or ascending colon on the right side, we may find in such cases, smaller or larger portions of these intestines spread over the surface of the cyst longitudinally and transversely, just the same as the Fallopian tube. It may be difficult, almost impossible, to remove the cyst-wall from the intestines in such cases, and danger may arise from the fact that the intestines will not bear denudation of the muscular layers to any extent, as it easily becomes gangrenous.

The first case I met with was that of a married woman, 22 years of age, from Racine, who had a cyst which had been developing for two years. It was as large as a gravid uterus at term and contained a clear fluid. When the abdomen had been opened and the covering broad ligament had been incised down to the cyst-wall, I commenced dissection with a view to enucleation, but after working about half an hour dissecting and ligating vessels, I had advanced but very little. All that I could get out of the cyst was a piece as large as the palm of the hand. Consequently I was obliged to leave the cyst, after having united the opening into it with the abdominal wound and made use of a method of drainage which I had intended to speak of this evening—the so-called Mikulicz drainage. The patient made a good recovery.

About a year ago, Mikulicz, of Cracow, proposed the following method of drainage, not only for retroperitoneal cysts, which can be excluded from the general peritoneal cavity by uniting them to the abdominal wound, but also for drainage in the peritoneal cavity itself. He takes a small piece of iodoform gauze, stitches a silk thread to the center of it, and folds it up in the form of a pouch, the silk thread being inside, so that the pouch may be drawn up from the bottom by it. The pouch is now pushed down to the bottom of the cavity, and, if nooks and corners exist, it is pushed out so as to completely fill them. In the inside of the pouch is packed with strips of idoform gauze, as much as is necessary to completely fill up these spaces. This is the advantage claimed by Mikulicz for his method of drainage as compared with the use of glass or rubber drainage.

DR. ETHERIDGE: Do you tuck that clear into the abdominal cavity—the cyst itself ?

Dr. FENGER: Yes sir, down into the bottom. Besides the disinfectant properties of the iodoform gauze applied to the entire wall of such a cyst Mikulicz states as one of the advantages of his method that, by the capillarity of the gauze, everything is brought out—fluids which a glass or rubber drain could not bring out. We must remember that when we drain the peritoneal cavity with a glass drain down between the intestines or in the cavity of the cyst, we cannot always expect to get surrounding organs in so close contact with the drain as to drive the fluid out.

Further, there is this to consider; that a glass drain put down in the free peritoneal cavity has no tendency to bring out the fluid accumulated at the bottom. The intestines filled with air will simply swim in the fluid and there is no pressure from without that will bring this fluid out of the glass drain, while the capillarity of the gauze is likely to help in that direction.

DR. SAWYER: How long could it be allowed to remain in the abdominal cavity ?

DR. FENGER: I have had it remain in all these cases for about two weeks. As soon as the discharge ceases I commence first to pull out the loose gauze inside the sack. If a space is left after this has been pulled out 1 press in at that dressing a little more gauze. This is gradually removed and the pouch itself is then pulled out by the thread gradually and finally. In all my cases it came out about the end of the second week.

The second case was similar to the first, inasmuch as there was no possibility, at least, as far as my ability went, of getting the cyst out. It was a large cyst of eight years' development, in a woman fifty years of age, from Sioux City, Iowa. A prolapse of the uterus had developed during this time and I was able to get out of the cyst, after considerable dissection, hardly more than two square inches. I used the Mikulicz drain with the same result as before. The patient was operated upon October 31, 1886.

In the two above mentioned cases enucleation was impossible, and we, with Ohlshausen, may have to class them under unfinished operations, as far as the extirpation of the cyst is concerned. But in cysts of the broad ligament, such an unfinished operation is, as a rule, followed by undisturbed and perfect recovery, and so I feel inclined rather to classify the above named method of operating as a legitimate one for non-enucleable parovarian cysts, than to use the somewhat misleading and sinister term of incomplete operation.

The third case was a woman 50 years of age, in whom the cyst had taken three years to develop. The operation was performed February 2, 1887. The outside of the cyst looked smooth in this case because the connective tissue was so loose. It was the easiest thing imaginable to enucleate it from the retroperitoneal cavity in which it was developed. There were not two vessels to tie and this accounts for the smoothness of the outer surface. This cyst was a typically normal one of that class, as it was covered all over with the broad ligament. The fluid was perfectly clear; no remnant of a blood clot was present.

Now, when the cyst has been enucleated, the question arises what to do. I was afraid to leave this large retroperitoneal wound without drainage, so I used Mikulicz' method, and the woman is well. It is, however, a debatable question ; and in the future it is probable that in a case like this drainage will not be used.

Authorities like Ohlshausen very strongly recommend, even for a cavity as large as that, not to drain at all—not even to unite the surface of the peritoneum so as to exclude the retroperitoneal wound from the general peritoneal cavity. He says that when there is no infection, no sepsis, during the operation, there will be no peritonitis, and no septicæmia afterward. He also states that he usually leaves the cavity alone after these enucleations, and that peritonitis seldom or never follows as a consequence of the operation, nor do pelvic abscesses form.

This is where the matter stands, and these are the points for discussion. I must say that I do not dare to rely so fully on entire asepsis during the operation as to leave drainage out. Undoubtedly the recovery of the patient is quicker and easier without than with drainage, as very often, in the latter case, a fistula remains which may keep open for months.

The fourth case was an old and rather anæmic patient, more than fifty years of age, but apparently sixty. She was pale and emaciated, and had a large retroperitoneal cyst, located partly in the peritoneal and partly in the retroperitoneal cavity, or, in other words, of partly extra and partly intra-ligamentous development. As a natural consequence, the enucleation was difficult, since the peritoneal cavity was at once entered. On the inside of the cyst were papillomatous masses such as are found in smaller growths, cystomas of the ovary. These, of course, always indicate malignancy. On the inside of this cyst the surface was rough, velvety from the diffuse papillomatous condition of the entire inner wall, and in some places grown out into a large papilloma, but in no place smooth.

The operation in this case was rendered more difficult, because the connective tissue, surrounding the intra-ligamentous portion of the cyst, was comparatively tense, and, further, because it had grown up into the mesentery of the sigmoid flexure, so as to be covered by it. When the cyst was enucleated, there was a portion of the sigmoid flexure that I was afraid of.

There is one other point beside the intestines which we should be careful to avoid in the extirpation of these retroperitoneal cysts; that is, the ureters. As soon as we get into the neighborhood of the large vessels in the posterior wall, we must look carefully out for the ureters and locate them by palpation, as when the ureter is adherent to the cyst it may be easily torn.

Mikulicz' drainage was used in this case as in the others. The first three or four days she had no untoward symptoms, but on the fourth or fifth day she commenced to vomit, and became somewhat delirious and sleepy, and died, the temperature not having exceeded 101° or 102° F. I saw her the evening before she died, and expected on account of the vomiting to find peritonitis, but there were no local symptoms at all. Then I supposed it to be sepsis without peritonitis, but the autopsy showed the cause of death to be uræmia.

We found in both kidneys, from pressure of the tumor on the ureters in a state of dilatation, not exactly hydronephrosis but dilatation and subsequent atrophy, to a sufficient degree in my opinion to account for uræmia; for we know that patients with so much degenerative disease of the kidneys of any kind as to almost reach the limit of secreting tissue, are apt to get uræmia after operation. Whether the operation, or the anæsthetic is the cause, I cannot say, but it is a well-known fact.

After the opening of the abdominal cavity, Mikulicz' drain was laid down right between the loops of intestine, and, of course, a local but aseptic peritonitis formed along the drain. You will notice on the specimen I now present the impression of the meshes of the tissue of the drain, but outside of this a perfectly clear and smooth peritoneum.

As I remarked before, the chief point for discussion is the drainage. Ohlshausen does not drain in any such cases. This may be thus explained: He says that in many cases of this kind it is impossible to finish the operation. If we accept his classification, two of my cases would be termed unfinished operations; but I am certain that with an unfinished operation and a Mikulicz's drain a radical cure may be effected just as well, perhaps, as if the cyst had been taken out. This, of course, would apply only to a thin-walled cyst of a malignant character.

DISCUSSION.

DR. A. REEVES JACKSON: If I remember correctly, Dr. Miner's discovery or suggestion in regard to enucleating tumors was not originally for the purpose of removing only sub-peritoneal cysts, but to make possible the removal of ovarian and other tumors in which the adhesions were so extensive as to make it impossible to remove them in any other way. I think his first case one in which he failed to find a pedicle and so was driven to enucleate. I think he removed very few tumors in this way, because he did not find the necessity for doing it. But when adhesions were so extensive and tense that it was impossible to remove the growth, he made a slight incision around its lower portion, and then, seizing it above, after emptying the cyst, he passed a finger or suitable instrument around until he had loosened all the attachments. It was a valuable suggestion, and operators have availed themselves of it in suitable cases.

In regard to the removal of these sub-peritoneal cysts, one of the cases related by Dr. Fenger shows clearly that the operation of enucleation is not always necessary. The tapping was followed by complete cure. Tapping has frequently been successful and these are the cases in which it is the proper method of treatment. I am aware that many hold a different opinion and advise extirpation always. If the tumor should refill after tapping and the patient's health fail, I should be in favor of removing the cyst by ordinary methods.

In regard to Mikulicz' plan of drainage, it has always seemed to me that it could only succeed in removing the thinner parts of the fluid; the more dense constituents could not be carried away as well by this method as by a tube of glass or rubber. I can see an objection to it in the possibility of a long convalescence resulting from the large fistulous opening which might be left, and which would be embarrassing for months.

In the last case detailed by Dr. Fenger, we have an illustration of the importance of knowing the condition of the kidneys before operating for any abdominal tumor of long standing. Many operators make it a point to know that the patient passes a sufficient quantity of urine of a proper character before consenting to operate. They insist on knowing that the kidneys can do their work, and the rule is a good one.

DR. CHRISTIAN FENGER: In regard to the remarks of Dr. Jackson, about the insufficiency of the Mikulicz' drain, I will say that I forgot to state that I do not use it now, without at the same time inserting in the center of it a glass or rubber drain. In an operation last Autumn, in which I used the Mikulicz drain without a glass drain, I found the outside dressing dry and still about a pint of fluid at the bottom of the abdominal cavity after the patient was dead; so since that time I have always inserted a glass or rubber drain in the center of the Mikulicz drain.

As to the remark regarding the examination of urine: Of course we always do this. In this case there was no albumen, but I cannot say that it was examined as to quantity.

In reply to the question of Dr. Jaggard as to whether I have used the drain in pelvic abscess and acute suppurative peritonitis: I have not used it in pelvic abscess, but have used it in tuberculous peritonitis, in a case last fall which looked like a tumor. There was tuberculous peritonitis with a large localized cavity or space filled with a fluid more or less serous. I used the Mikulicz drain with a glass drain, and the patient was still alive two or three months ago, and I do not know whether the drain is out yet. I have used it in other cases which show its efficiency. In a case of chylous ascites that had been tapped and tapped and had refilled, and in which laparotomy had been performed the year before and still it had filled again, I opened and drained the peritoneal cavity, and used the Mikulicz drain with a glass tube with perfect success. I always use it in acute peritonitis, and see no reason why it should not be used in peritonitis as well as in other cases. At the time Mikulicz published his paper on this form of drainage, it was only in peritonitis that he had used it.

In regard to the similarity between labor and operation, as far as the question of eclampsia is concerned: That is one of the subjects which was brought up for discussion in the *Berliner Kleinsche Medicinische Gesellschaft* last fall, between Leyden and Virchow, in which no definite conclusion was reached.

I fully agree with Dr. Etheridge that vaginal drainage, mechanical of course, is the most rational method because it draws best. My own experience is limited as I have only employed it twice, and both patients died. We dread the vagina as a septic cavity and the reason why we should not drain through it, is fear of sepsis from the vagina up into the abdominal cavity. But I believe with our new precautions the vagina could be kept so aseptic that retrograde sepsis from it could be prevented. I believe also that vaginal drainage ought to be used more than it is, and that we need not be so afraid of it as we are.

W. W. JAGGARD, M. D., Editor.

2330 Indiana avenue.

